# Prevalence and genetic characteristics of lincosamide resistance genes lsa(E) and lnu(B) in group B Streptococcus from southern China

**DOI:** 10.1099/mgen.0.001482

**Published:** 2025-09-16

**Authors:** Chang-Song Wu, Jian-Hao Lin, Ming Chen, Yan Huang, Yong-An Zhang

**Affiliations:** 1 National Key Laboratory of Agricultural Microbiology, Hubei Hongshan Laboratory, Engineering Research Center of Green Development for Conventional Aquatic Biological Industry in the Yangtze River Economic Belt, Ministry of Education, College of Fisheries, Huazhong Agricultural University, Wuhan, PR China; 2 Guangxi Zhuang Autonomous Region Center for Disease Control and Prevention, Nanning, PR China

**Keywords:** antibiotic resistance, group B *Streptococcus*, *lsa(E)*, *lnu(B)*, mobile genetic elements

## Abstract

Lincosamides serve as alternative therapeutic agents for penicillin-allergic patients with *Streptococcus agalactiae* [group B *Streptococcus* (GBS)] infections, but the escalating antibiotic resistance has severely compromised their clinical efficacy. This study investigated the epidemiological characteristics of antibiotic resistance and the co-transfer mechanisms of lincosamide resistance genes *lsa(E)* and *lnu(B)* in 631 clinical GBS isolates from southern China. The results showed that 98.3% of isolates carried at least one antibiotic resistance gene, with lincosamide resistance genes detected in 76.1% of isolates. The *lsa(E)* and *lnu(B)* were identified as prevalent lincosamide resistance determinants alongside *erm(B)*, with 18.7% of isolates co-carrying *lsa(E)* and *lnu(B)*, and double-positive isolates predominantly distributed in high-risk clonal complexes (CCs): CC10 (15.4%), CC17 (21.7%), CC19 (17.9%) and CC103 (35.5%). Genomic analyses revealed that *lsa(E)–lnu(B)* formed composite resistance modules by integrating into resistance gene clusters within integrative and conjugative elements (ICEs), with insertion sequence-mediated mobilization increasing their dissemination risk. In CC10 and CC17, ICE*Sag37* served as the primary ICE harbouring *lsa(E)–lnu(B)*, while novel genetic contexts for *lsa(E)–lnu(B)* were identified in CC19, CC23 and CC103. This study highlights the high prevalence of *lsa(E)–lnu(B)* resistance clusters in GBS clinical isolates from southern China and their ICE-mediated dissemination mechanisms, providing critical molecular epidemiological evidence for resistance surveillance and precision therapeutic strategies.

**Impact Statement**

This study provides insight into the genetic context and transmission dynamics of the lincosamide resistance genes *lsa(E)* and *lnu(B)* in clinical GBS isolates from southern China, revealing the high prevalence of the *lsa(E)–lnu(B)* resistance cluster and its integrative and conjugative element (ICE)-mediated transmission mechanisms. The findings highlight the risk that ICE-driven spread of multidrug resistance modules may trigger regional or even global public health crises, necessitating urgent interventions through molecular surveillance, targeted therapies and enhanced antibiotic stewardship. These results not only provide a theoretical basis for optimizing clinical treatment strategies but also lay the groundwork for developing interdisciplinary prevention and control policies, contributing profoundly to safeguarding public health security.

## Data Summary

Genome sequences of 631 clinical isolates have been deposited into the National Center for Biotechnology Information database under the BioProject accession number PRJNA1046429. The authors confirm that all supporting data, code and protocols have been provided within the article or through supplementary material.

## Introduction

*Streptococcus agalactiae*, or group B *Streptococcus* (GBS), is a major pathogen responsible for neonatal invasive infections and perinatal complications in pregnant women [1]. In pregnant women, common manifestations of GBS include bacteraemia, urinary tract infections, chorioamnionitis and premature rupture of membranes. These conditions are the underlying cause of adverse events such as preterm birth and stillbirth [2]. Although penicillin remains the first-line treatment for GBS infections, patients allergic to *β*-lactam antibiotics rely on alternative therapies such as lincosamides (clindamycin) and macrolides (erythromycin) [3]. However, the global prevalence of GBS resistance to clindamycin and erythromycin has been escalating in recent years, posing a cross-regional public health threat [4]. According to global surveillance data, resistance rates to clindamycin and erythromycin are 43.2% and 54.8% in the USA [5], 26.0% and 23.8% in Denmark [6], 5.2% and 25.9% in Brazil [7], 26.0% and 25.0% in Argentina [8] and 3.8% and 16.1% in South Africa [9], respectively. The antimicrobial resistance situation in China is particularly serious. Data from the China Antimicrobial Resistance Surveillance Network in 2021 showed that resistance rates to clindamycin and erythromycin reached 59.7% and 74.5%, respectively, with clindamycin resistance exceeding 70% in Liaoning and Hebei provinces [10,11]. The rapid spread of antimicrobial resistance not only threatens individual patient outcomes but also raises concerns about broader public health implications due to the potential for regional or global dissemination of resistant strains.

The primary driver of GBS resistance is the horizontal transmission of acquired antibiotic resistance genes (ARGs) [12]. Among these, *erm* family genes are the dominant contributors to the macrolide–lincosamide–streptogramin B (MLSB) resistance phenotype. The *erm* genes encode ribosomal methyltransferases that modify the target site on 23S rRNA, reducing antibiotic binding efficiency and conferring cross-resistance to MLSB drugs [3,13]. However, recent studies have highlighted the emergence of non-*erm*-dependent lincosamide resistance mechanisms, particularly the synergistic action of the *lsa(E)* and *lnu(B)* genes [14]. The *lsa(E*) gene encodes an ABC-F protein that directly counteracts lincosamide inhibition via ribosomal protection, while the *lnu(B)* gene encodes a nucleotidyltransferase that chemically modifies lincosamides to inactivate them [3,15]. Compared to the widespread distribution of *erm* genes in GBS, *lsa(E)* and *lnu(B)* appear to be rare and have been reported in the USA, South Korea and China [12,16, 17]. Notably, *lsa(E)* and *lnu(B)* frequently coexist with resistance genes for tetracyclines, macrolides and aminoglycosides within the same mobile genetic element, forming composite resistance modules. Genes within composite resistance modules are collectively expressed through the integron promoter, which enables the bacterial strain to acquire a multidrug resistance phenotype and constitutes an essential mechanism for maintaining multidrug resistance in dynamic environments [18,20]. This genetic colocalization not only enhances multidrug resistance but also accelerates the spread of resistance within bacterial populations through horizontal gene transfer [21].

This study systematically analysed the antibiotic resistance profiles of 631 GBS clinical isolates from southern China, with a focus on the epidemiological characteristics and genetic contexts of the *lsa(E)–lnu(B)* resistance cluster. Through genomic analysis, we revealed the distribution patterns of *lsa(E)–lnu(B)* across clonal complexes and identified its integration mechanisms within integrative and conjugative elements (ICEs). Furthermore, we evaluated the association between this resistance cluster and other resistance determinants to analyse its transmission mechanism in GBS populations. These findings provide the basis for developing targeted therapeutic and control strategies against GBS infections.

## Methods

### Clinical GBS isolates and whole-genome sequences

The 631 GBS isolates collected from the Guangdong and Guangxi provinces in southern China between 2018 and 2021, as analysed in our previous study [22], were used in this study. These isolates were isolated, cultured and identified by local hospital laboratories from patients. Detailed information on these isolates is provided in Table S1 (available in the online Supplementary Material).

### Multilocus sequence typing, serotyping and antibiotic resistance gene detection

Multilocus sequence typing (MLST) of all isolates was performed using mlst (v2.22.1) based on seven housekeeping genes (*adhP*, *pheS*, *atr*, *glnA*, *sdhA*, *glcK* and *tkt*) [23]. The sequence types (STs) were determined according to the allelic profiles subsequently. The clonal complexes (CCs) were analysed using goeBURST (v1.2.1) based on single locus variant [24]. The capsular serotypes were analysed using GBS-SBG (https://github.com/swainechen/GBS-SBG) [25]. ARGs were detected using ABRicate (v1.0.0) (https://github.com/tseemann/abricate), and genes were considered present if there was 80% coverage at 80% nucleotide identity [26].

### Phylogenetic analysis

Whole-genome alignment was performed using Snippy (v4.6.0) (https://github.com/tseemann/snippy) with BM110 (GenBank accession number GCA_900155855.1) as the reference genome. Recombination was detected and removed using Gubbins (v3.1.3) [27]. A recombination-free phylogeny was produced by RAxML (v8.2.12) with the GTRGAMMA model and then visualized using iTOL (v6.7.6), together with associated metadata [28,29].

### Identification of resistance gene cluster *lsa(E)*–*lnu(B)* and mobile genetic elements

Genome sequences were mapped against the genomic fragment containing the *lsa(E)* and *lnu(B)* genes using a pipeline based on the Mummer (v3.1) package, and the gene cluster was considered present if there was 90% coverage at 90% nucleotide identity [30]. Mobile genetic elements (MGEs) were predicted using VRprofile (v2.0) and ICEberg (v2.0) databases [31,32]. Sequence alignments of MGEs were performed using Easyfig (v2.2.5) [33].

### Statistical analysis

Statistical analysis was performed using GraphPad Prism (v8.0.2). ANOVA test with Tukey multiple comparison test was used to determine significant differences in the number of ARGs between isolates carrying the *lsa(E)* and *lnu(B)* genes and those not carrying. *P*-value<0.001 indicated statistical significance.

## Results

### Epidemiological characteristics of GBS antimicrobial resistance in southern China

Among the 631 GBS isolates from southern China, 25 different antibiotic resistance determinants were identified. The number of ARGs harboured by different CC isolates varied, such as CC17 isolates harbouring the highest number of ARGs (median: 7) and the lowest in CC1 (median: 2) (Fig. S1A). 98.3% (620/631) of isolates harboured at least one ARG, including 14.1% (89/631) with two ARGs and 64.5% (407/631) with three or more ARGs (Fig. S1B). Of these, 14.1% (89/631) of isolates harboured ARGs against a single antibiotic class (84 isolates were tetracycline-resistant), 1.9% (12/631) exhibited dual-class resistance (tetracyclines, aminoglycosides or phenicols) and 82.3% (519/631) were multidrug-resistant (MDR) with ARGs against three or more antibiotic classes.

**Fig. 1.**
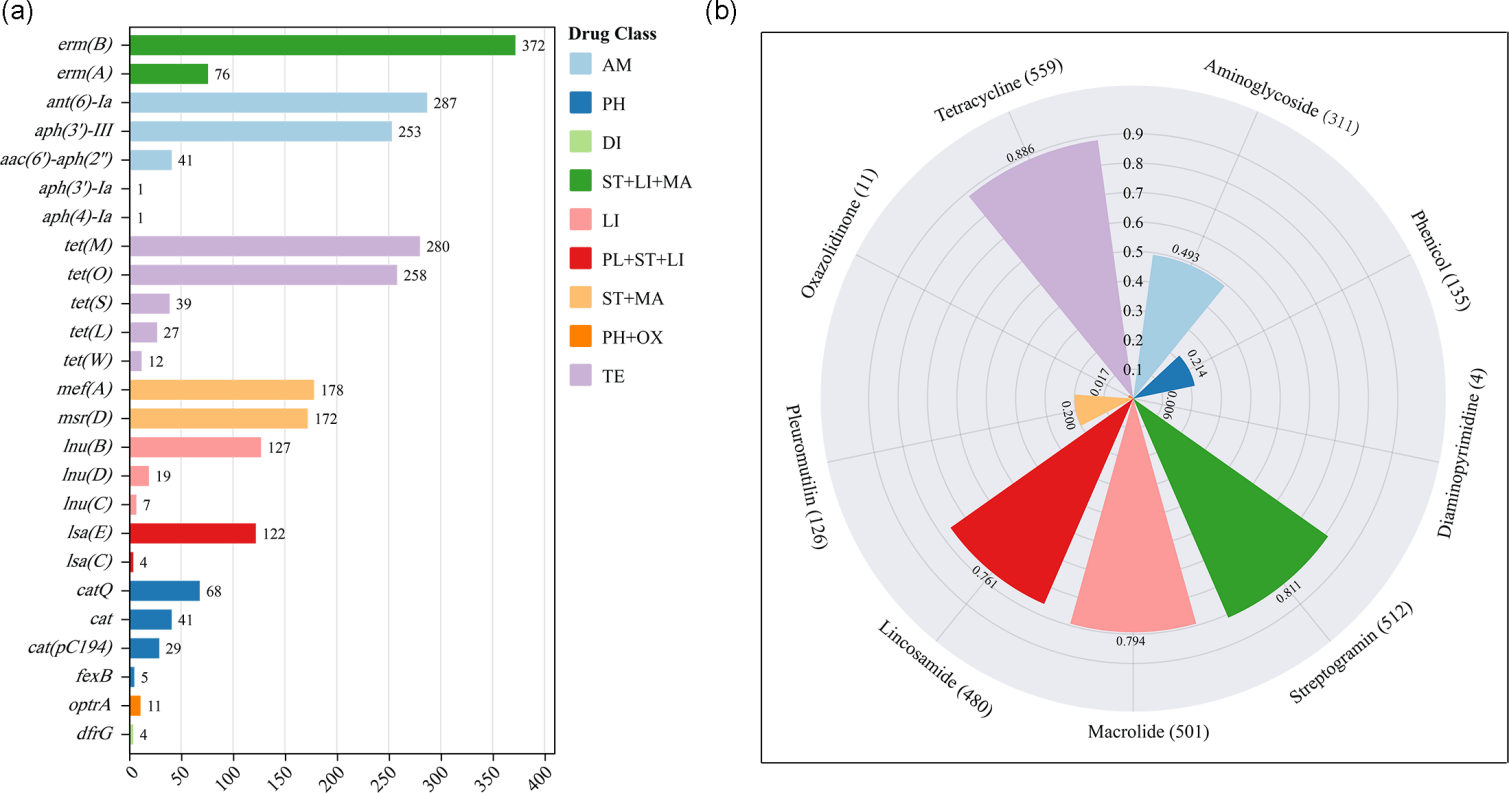
Distribution of ARGs and antibiotic resistance profiles in 631 isolates. (**a**) Abundance and resistance classification of ARGs in 631 isolates. ARGs are coloured according to antibiotic resistance and resistance combinations. AM, aminoglycoside; PH, phenicol; DI, diaminopyrimidine; ST, streptogramin; LI, lincosamide; MA, macrolide; PL, pleuromutilin; OX, oxazolidinone; TE, tetracycline. (**b**) Antibiotic resistance profiles of 631 isolates. The frequency of each antibiotic resistance is indicated.

In this investigation, *erm(B*) was the most prevalent ARG, detected in 59.0% (372/631) of isolates, followed by *ant(6)-Ia* (45.5%, 287/631) and *tet(M*) (44.4%, 280/631) (Fig. 1a). Tetracycline resistance determinants were identified in 88.6% (559/631) of isolates, with *tet(O*) (40.9%, 258/631), *tet(S*) (6.2%, 39/631), *tet(L*) (4.3%, 27/631) and *tet(W*) (1.9%, 12/631) being additionally observed, including co-occurrence of two or three *tet* variants in some isolates. Resistance genotypes for streptogramins (81.1%, 512/631), macrolides (79.4%, 501/631) and lincosamides (76.1%, 480/631) were also highly prevalent, reflecting the ability of distinct ARGs to confer overlapping resistance phenotypes and the multifunctional nature of certain resistance determinants (Fig. 1b) [3]. Notably, 73.7% (465/631) of isolates simultaneously harboured resistance determinants for streptogramins, macrolides and lincosamides, a phenomenon strongly associated with the combinatorial distribution of these ARGs.

### Distribution of *lsa(E)* and *lnu(B)* genes in different lineages

**Fig. 2.**
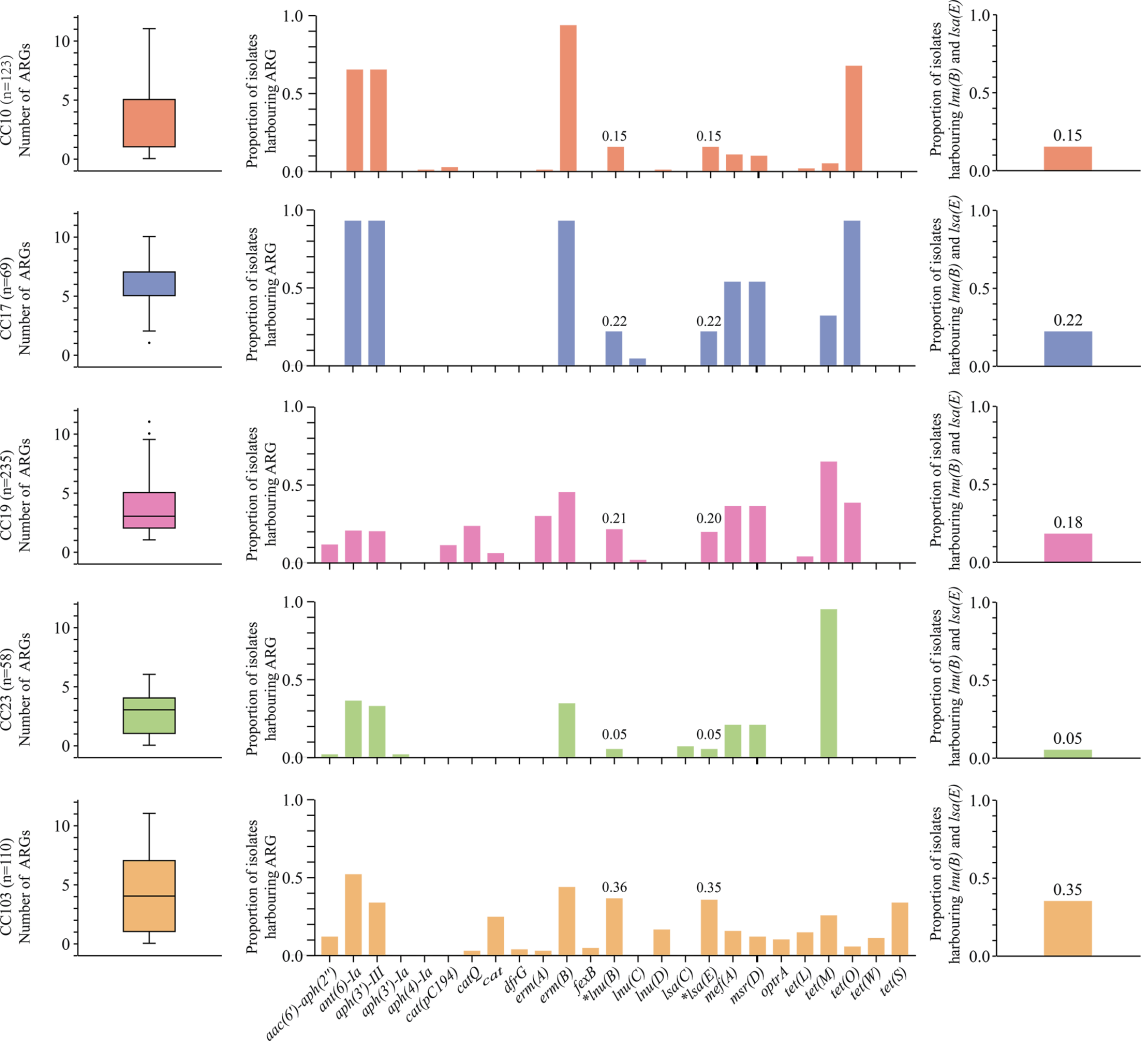
Distribution of ARGs in lineages containing *lsa(E*) and *lnu(B*). Left: Box and whisker plot of the number of resistance genes carried by each CC. Middle: The proportion of isolates from each CC carrying any specific resistance gene. Right: The proportion of isolates from each CC harbouring *lsa(E*) and *lnu(B*).

The *lsa* and *lnu* family genes were detected in 24.1% (152/631) and 20.0% (126/631) of isolates, respectively, predominantly distributed among CC10, CC17, CC19, CC23 and CC103 lineages (Fig. 2). The *lsa(E*) (96.8%, 122/126) and *lnu(B*) (83.6%, 127/152) emerged as the predominant variants of the *lsa* and *lnu* genes, with the highest prevalence observed in CC103, followed by CC17 and CC19. Notably, *lsa(E*) and *lnu(B*) exhibited consistent co-occurrence across isolates (co-existing in 118 isolates), except eight CC19 isolates carrying *lnu(B*) alone, four CC19 isolates carrying *lsa(E*) alone and one CC103 isolate carrying *lnu(B*) alone. Additionally, uncommon *lsa* and *lnu* variants were identified: *lnu(D*) was detected in 18 CC103 and 1 CC10 isolates, *lnu(C*) in 3 CC17 and 4 CC19 isolates and *lsa(C*) exclusively in 4 CC23. Except for a single isolate harbouring the *lnu(D)–lnu(B)–lsa(E*) combination, these rare variants were carried in isolation without combinatorial distribution.

### Antibiotic resistance genotypic characteristics of *lsa(E)*- and *lnu(B)*-positive GBS isolates

**Fig. 3.**
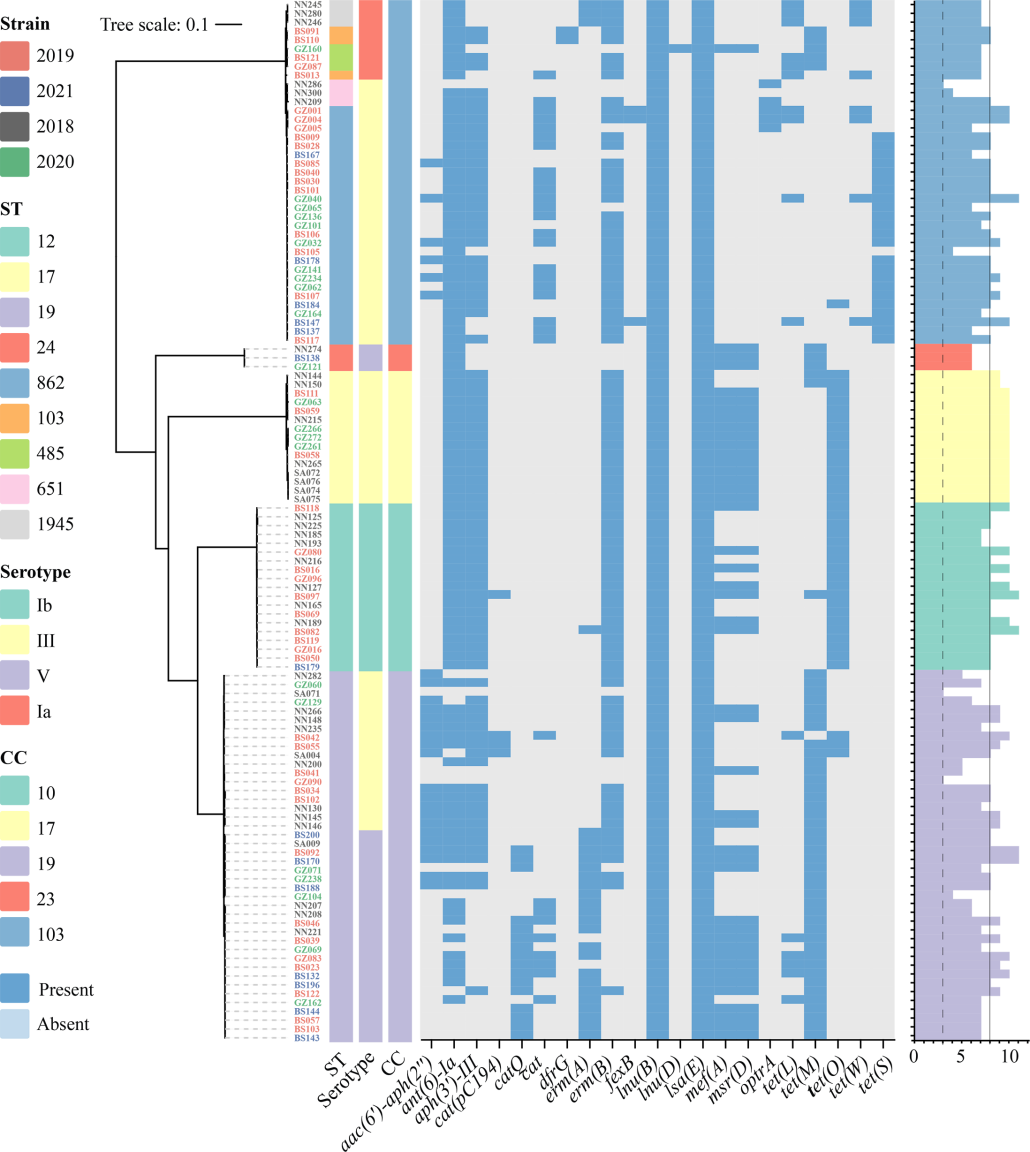
Distribution of ARGs in 118 isolates containing *lsa(E) *and *lnu(B)*. The tree (left) is coloured based on collection time. STs, serotypes and CCs are indicated. The blue blocks of the heatmap (middle) indicate the presence of ARGs, and the grey blocks indicate the absence. The number of ARGs carried by each isolate is represented by a bar chart (right). The solid line indicates the median number of ARGs. The scale bar shows the number of nucleotide substitutions per site.

The *lsa(E)* and *lnu(B)* genes were identified in 20.8% (131/631) of the isolates, with 90% (118/131) of these being double-positive for both genes, accounting for 18.7% of the total isolates. Double-positive isolates were distributed across CC10, CC17, CC19, CC23 and CC103 lineages at proportions of 15.4% (19/123), 21.7% (15/69), 17.9% (42/235), 5.2% (3/58) and 35.5% (39/110), respectively (Fig. 2). In CC10, CC17 and CC23, double positivity was exclusively associated with singular STs and serotypes, such as ST12 (Ib), ST17 (III) and ST24 (V) (Fig. 3). In CC19, double positivity was restricted to ST19 isolates, comprising 18 serotype III and 24 serotype V isolates. Compared to serotype III, serotype V isolates mainly carried *erm(A*), *cat(Q*) and *cat* genes. Double-positive isolates were detected in five STs of CC103, most of which were ST862 serotype III strains (*n*=27). Five tetracycline resistance determinants were identified in CC103, with the rare *tet(S*) being most prevalent and primarily associated with ST862. The phenol antibiotic resistance gene *cat* (*n*=22) was also abundantly distributed in CC103, and in addition, the rare *optr(A)* gene was detected in five isolates. Double-positive isolates demonstrated a higher median ARG burden (median: 8, Fig. S2), a phenomenon potentially linked to the co-localization of *lsa(E)* and *lnu(B)* with additional resistance gene clusters on MGEs.

### Genetic context of *lsa(E)*–*lnu(B)* in CC10 and CC17

The mobile element ICE*Sag37*, originally identified in Ib/ST12 isolates and subsequently reported in III/ST17 isolates [34,35], was detected in both CC10 and CC17 isolates harbouring *lsa(E)–lnu(B)*. ICE*Sag37* contains a two-component signal transduction system (*nisK*/*nisR*), *mobC*, integrase and relaxase genes, along with putative virulence factors and multiple ARGs [34]. Comparative genomic analysis showed that ICE*Sag37* replaced the ancestral genomic regions containing the pilus island PI-1 and the insertion sequence (IS) ISSag5 in ST12 and ST17 isolates (Fig. S3), a finding consistent with previously reported studies [34,35]. Distinctively, *lsa(E)* and *lnu(B)* were integrated into the resistance gene mosaic region of ICE*Sag37* in *lsa(E)–lnu(B)*-positive isolates, forming a novel resistance cluster: *tet(O)–ant(6)-Ia–lsa(E)–lnu(B)–aph(3′)-III–erm(B)*. Furthermore, an insertion of ISEfa5 (ISL3 family transposon) was identified downstream of *lnu(B)*, which has been reported to be widely distributed in *Enterococcus faecalis* and associated with mobile element-mediated antibiotic resistance [36,37].

### Multidrug resistance clusters harbouring *lsa(E)*–*lnu(B)* in CC19, CC23 and CC103

Three *lnu(B)–lsa(E*)-positive CC23 isolates exhibited identical antibiotic resistance genotypes, likely attributed to harbouring the same ICE. The novel ICE*Gz121* (accession no. PV832434), integrated into the chromosomal *comEC* locus – a recognized integration hotspot in GBS [22,38] – was identified in these isolates. This ICE contained an MDR cluster comprising five ARGs *[ant(6)-Ia–lsa(E)–lnu(B)–mef(A)–msr(D)*], with IS3 family transposons (ISStin6 and ISSdy2) inserted into the MDR region (Fig. S4A). In CC19, two distinct ICEs carrying MDR clusters were identified: one present in serotype III and eight serotype V isolates and another exclusive to the remaining serotype V isolates. The novel ICE*Sa009* (accession no. PV832435) predominant in serotype III isolates contained the MDR cluster *lsa(E)–lnu(B)–aac(6′)-aph(2″)–ant(6)-Ia–aph(3′)-III–erm(B)*, integrated between the chromosomal *uvrB* and *secA* loci (Fig. S4B). The serotype V associated ICE (accession no. MK102985), previously reported in CC19, harboured *tet(L)–cat–ant(6)-Ia–lsa(E)–lnu(B)* [17]. Both ICEs featured IS6 and IS3L family transposons flanking *lsa(E)–lnu(B)*, likely contributing to modular rearrangements and resistance gene loss during clonal dissemination [21,39]. Five STs were identified in CC103, with ST862 predominating (69.2%). The genomic location or putative ICE could not be determined in ST103, ST651 and ST862 due to the short length of the assembled contigs. The *lsa(E)–lnu(B)* locus resided on a truncated contig (11 kb), flanked downstream by ISEfa5 and upstream by *ant(6)-Ia* as the sixth gene. In ST485, novel ICEGz087 (accession no. PV832433) containing *lsa(E)–lnu(B)* was integrated between the *rplL* and *yxdL* genes, a known ICE insertion hotspot in GBS [12]. The MDR cluster *lsa(E)–lnu(B)–ant(6)-Ia–aph(3')-III–erm(B)-tet(L*) and six transposases were present in this ICE (Fig. S4C). In ST1945, *lsa(E)–lnu(B)* was located on the right side of a contig containing the MDR cluster *lsa(E)–lnu(B)–ant(6)-Ia–tet(W)–tet(L)*. The *tet(L)* gene was adjacent to an IS3 family transposase (IS861), but contig truncation at this site precluded full ICE characterization. However, the detection of the *rplL* gene upstream of *lsa(E)–lnu(B)* suggests potential ICE integration between *rplL* and *yxdL*, consistent with the insertion pattern observed in ST485.

## Discussion

Antimicrobial resistance in GBS is an increasingly serious threat to global public health. This study showed that GBS clinical isolates in southern China were generally multidrug-resistant, especially to tetracyclines, macrolides, lincosamides and streptogramins. Tetracycline resistance genes were detected in 88.6% of isolates (*tet(M)* and *tet(O)* predominating), aligning with global trends (81.6–94.6% in Europe [40,41], 83.9–86.0% in the Americas [5,7], 82.6% in Africa [42]). Resistance to macrolides (79.4%), lincosamides (76.1%) and streptogramins (81.1%) was primarily mediated by *erm* family genes (69.1%) in the present study, reflecting the dominance of the MLSB cross-resistance phenotype among GBS isolates in southern China. Notably, the resistance rates to macrolides and lincosamides were significantly higher than international levels and the national average [3]. In particular, the resistance rate to lincosamide antibiotics exceeded the national average by more than 15% [10]. In addition to *erm* genes, 24.1% and 20.0% of isolates harboured *lsa* and *lnu* genes, respectively, with 7.0% (44/631) carrying *lsa* or *lnu* genes independently of *erm*, contributing additional lincosamide resistance.

The widespread distribution of *lsa* and *lnu* family genes among clinical GBS isolates in southern China demands considerable clinical concern. In this study, *lsa(E)* and *lnu(B)* emerged as the predominant subtypes within their respective families, with 18.7% (118/631) of isolates harbouring both genes. The co-occurrence of *lsa(E)* and *lnu(B)* has been previously reported in *Streptococcus suis*, *Staphylococcus aureus* and *Listeria monocytogenes* [43,45], and their synergistic effect in GBS is believed to confer enhanced multidrug resistance [16]. Furthermore, studies have indicated that *lsa(E)* and *lnu(B)* are genomically adjacent loci and commonly integrated into ICEs containing multiple resistance genes [17,46]. ICEs serve as key vectors for horizontal gene transfer, integrating into the chromosome via site-specific recombination mediated by integrases and facilitating the transmission of resistance modules between hosts [21]. This study also showed that the distribution of the *lsa(E)–lnu(B)* cluster was highly correlated with ICEs. For instance, in CC10 and CC17, *lsa(E)–lnu(B)* was integrated into the resistance gene mosaic region of ICE*Sag37*, forming a novel composite resistance unit. The insertion of new resistance determinants undoubtedly exacerbates the clinical challenges in treating GBS infections. These findings underscore the critical role of ICEs in driving the evolution of multidrug resistance among GBS pathogens in southern China.

In addition, this study revealed that the *lsa(E)–lnu(B)* cluster exhibited different genetic contexts in CC19, CC23 and CC103, with distinct ICEs and multidrug resistance clusters observed among different STs and serotypes. The presence of multiple IS elements within the resistance regions suggests mobilization potential and increases the risk of IS-mediated insertion of conserved resistance modules [21,47]. The widespread distribution of *lsa(E)–lnu(B)* in CC103 may contribute to its recent expansion in southern China [22]. Notably, *tet(S)*, which is prevalent in bovine GBS isolates in China, remains rare in human GBS strains [48]. As an emerging zoonotic lineage, CC103 may facilitate cross-species resistance transmission under agricultural antibiotic selection pressures. The *optr(A)* gene, originally identified in *Enterococcus* species from animals and humans in China, encodes an ABC-F protein that confers resistance to phenicols and oxazolidinones through a ribosome protection mechanism [49]. Oxazolidinones are important treatment options for Gram-positive bacterial infections due to their clinical efficacy and favourable safety profile, particularly as a last-resort therapy for *Staphylococcus aureus* infections [50]. The detection of *optr(A)* in five *lsa(E)–lnu(B)*-positive CC103 isolates further underscores the risk of cross-species dissemination of resistance determinants. Therefore, strict monitoring of oxazolidinone use in the treatment of GBS infections is essential to prevent both intra- and interspecies resistance transmission.

However, this study also has some limitations. First, while we comprehensively characterized the genotypic antimicrobial resistance profiles of *lsa(E)* and *lnu(B)*-positive isolates, phenotypic susceptibility testing was not performed. The absence of *in vitro* resistance phenotype data limits our ability to functionally validate the contribution of these genes to observed resistance levels, particularly for rare genotypes. Second, reliance on short-read whole-genome sequencing hindered the complete resolution of MGEs. Fragmented assemblies in repetitive genomic regions limited precise characterization of resistance module boundaries and their chromosomal integration sites. Future studies integrating long-read sequencing and phenotypic validation will address these shortcomings and enhance clinical relevance.

This work systematically investigated the MDR characteristics of clinical GBS isolates from southern China, revealing the high prevalence of the *lsa(E)–lnu(B)* resistance cluster and its dissemination mechanism driven by MGEs. These genes form complex resistance modules through integration with ICEs and spread widely among high-risk clones. The clinical implications extend beyond lincosamide resistance, as the genetic co-transfer of these elements may further exacerbate the multidrug resistance crisis. Addressing this challenge requires multidisciplinary collaboration integrating genomic surveillance, precision therapeutics and enhanced antibiotic stewardship to curb the spread of antimicrobial resistance and safeguard public health security, particularly in high-burden regions such as southern China.

## Supplementary material

10.1099/mgen.0.001482 Uncited Fig. S1.

10.1099/mgen.0.001482 Uncited Table S1.

## Abbreviations

ARG: antibiotic resistance gene
CCs: clonal complexes
GBS: group B *Streptococcus*
ICEs: integrative and conjugative elements
IS: insertion sequence
MDR: multidrug-resistant
MGEs: mobile genetic elements
MLSB: macrolide–lincosamide–streptogramin B
MLST: multilocus sequence typing
STs: sequence types
