# Supplementary material for: Prevalence and genetic characteristics of lincosamide resistance genes lsa(E) and lnu(B) in group B Streptococcus from southern China
Source: Microb Genom. 2025 Sep 16;11(9):001482. doi: 10.1099/mgen.0.001482 (PMC12441131; doi:10.1099/mgen.0.001482)
Supplement: Uncited Fig. S1. [file mgen-11-01482-s001.pdf]

**Supplemental Material**

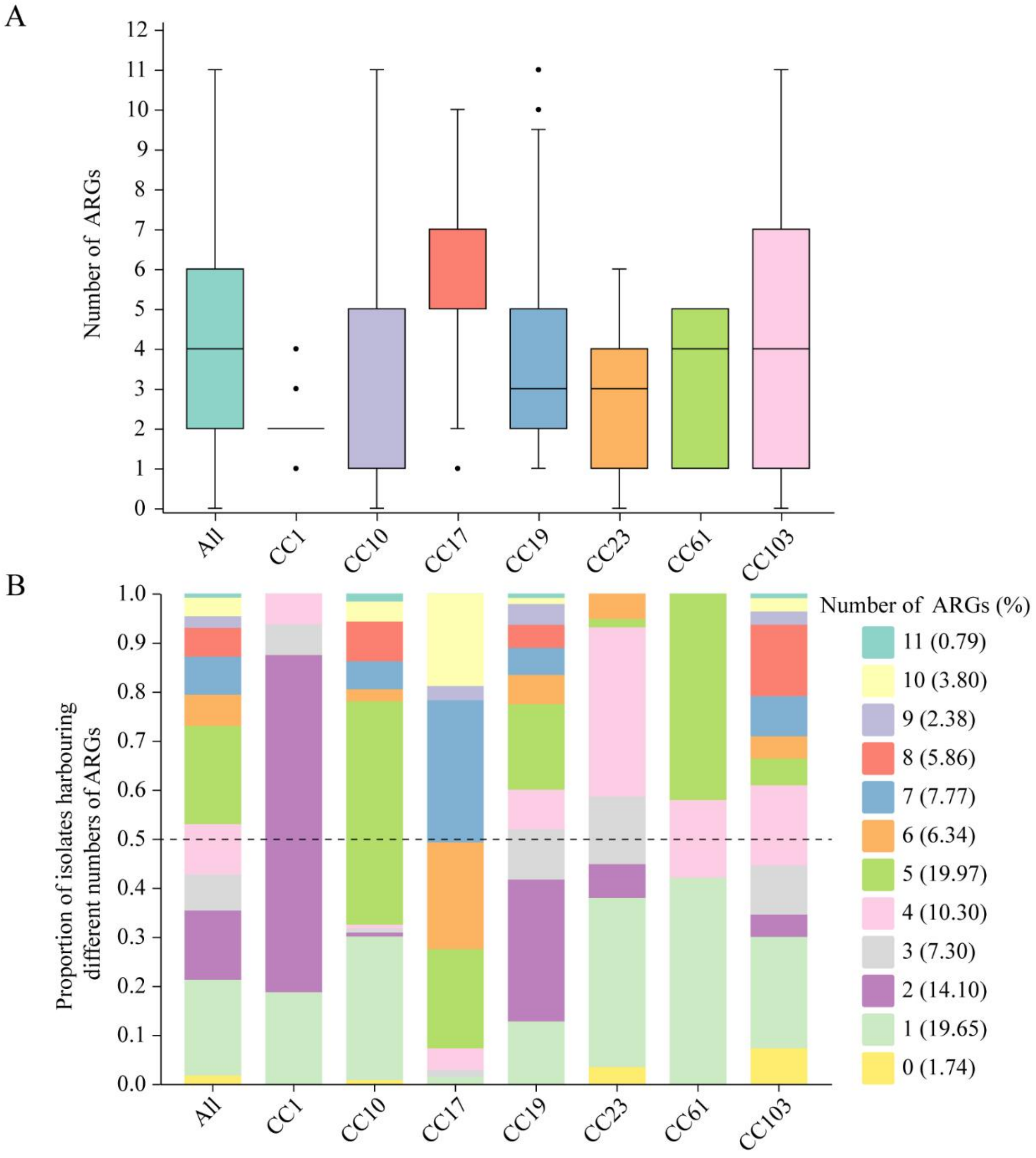


**Figure S1. Distribution of ARGs in lineages.** (A) Box and whisker plot of the number of resistance genes carried by each CC. (B) The proportion of isolates from each CC carrying a specific number of resistance genes. The dotted line indicates the median number of resistance genes.

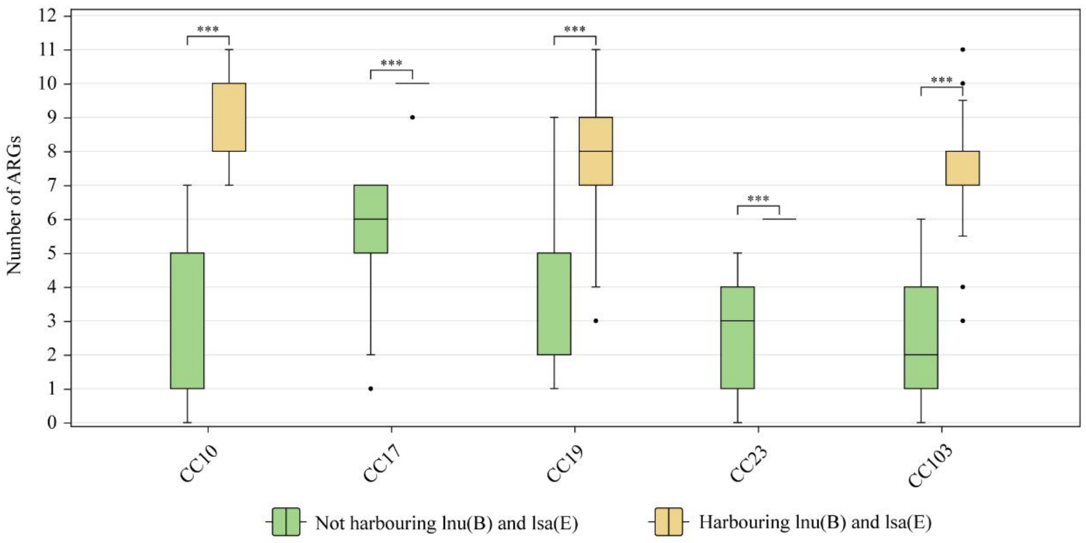


**Figure S2. Comparison of isolates carrying and not carrying *lsa(E)* and *lnu(B)* within lineages.** The asterisk indicates significant difference (***$p < 0.001$).

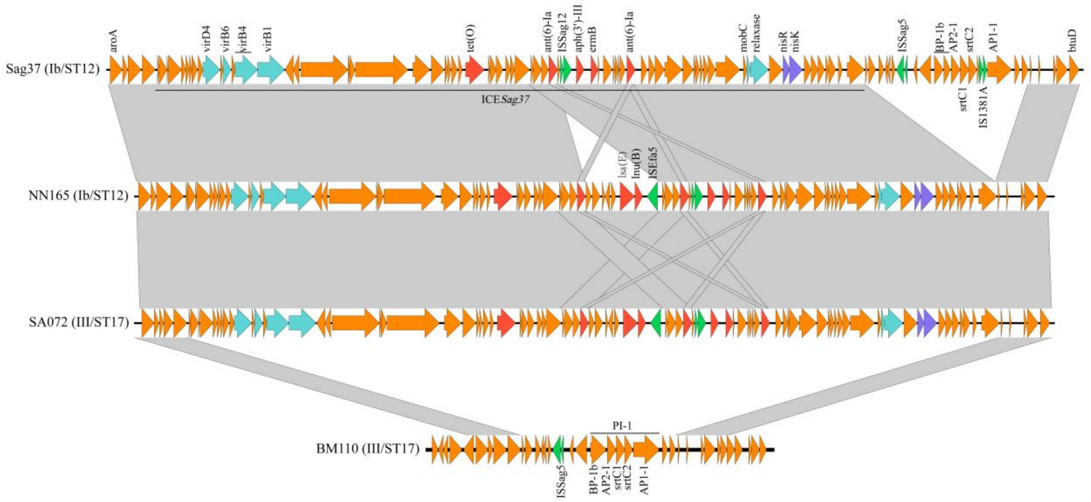


**Figure S3. Genetic context of *lsa(E)–lnu(B)* in CC10 and CC17.** The *lsa(E)–lnu(B)* locus is integrated into the multidrug resistance region of ICE*Sag37*. The direction of the arrow indicates the direction of transcription. Homologous regions are shaded in gray. ARGs are labeled in red, insertion sequences in green, putative virulence factors in sky blue, and signal transduction systems in purple.

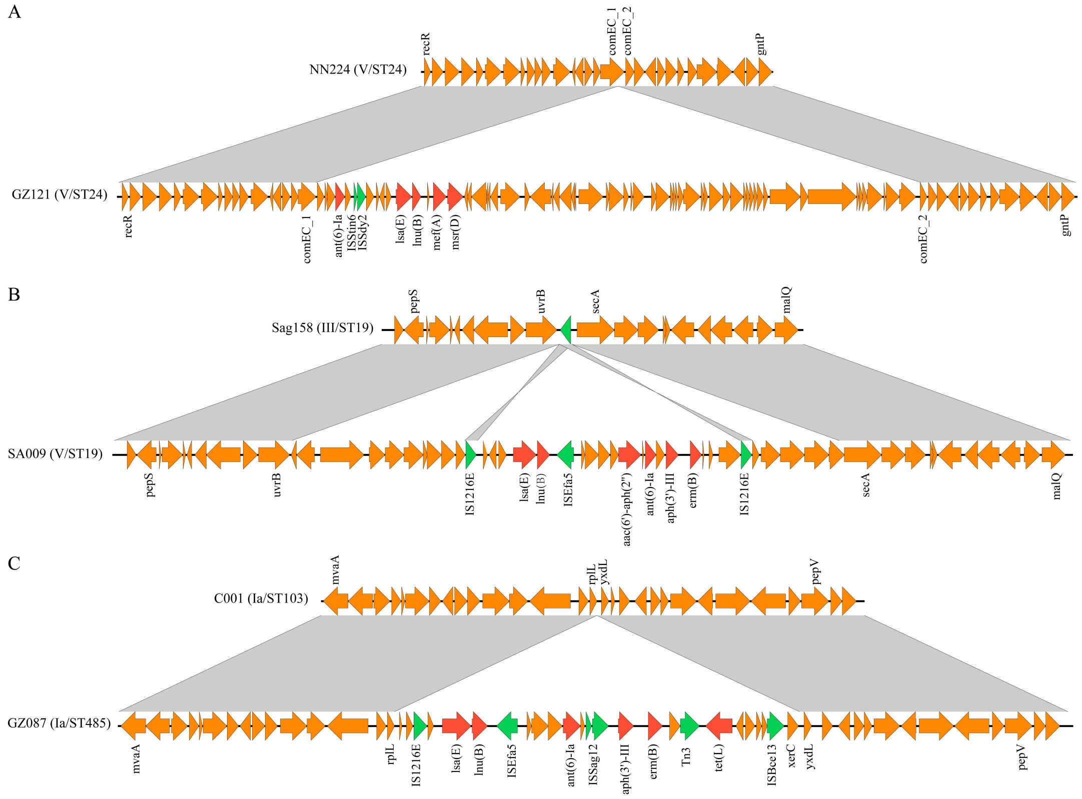


**Figure S4. Genetic context of *lsa(E)–lnu(B)* in CC23 (A), CC19 (B) and CC103 (C).** The direction of the arrow indicates the direction of transcription. Homologous regions are shaded in gray. ARGs are labeled in red and insertion sequences in green.
